# Effect of a single initial phase of non-surgical treatment of peri-implantitis: Abrasive air polishing versus ultrasounds. A prospective randomized controlled clinical study

**DOI:** 10.4317/jced.56653

**Published:** 2020-10-01

**Authors:** Amparo Aloy-Prósper, Hilario Pellicer-Chover, David Peñarrocha-Oltra, Miguel Peñarrocha-Diago

**Affiliations:** 1 Assistant Professor of Oral Surgery, Stomatology Department, Faculty of Medicine and Dentistry, University of Valencia, Valencia, Spain; 2 Collaborating Professor of the Master in Oral Surgery and Implant Dentistry, Stomatology Department, Faculty of Medicine and Dentistry, University of Valencia, Valencia, Spain; 3 Doctor Assistant of Oral Surgery, Stomatology Department, Faculty of Medicine and Dentistry, University of Valencia, Valencia, Spain; 4 Chairman of Oral Surgery, Stomatology Department, Faculty of Medicine and Dentistry, University of Valencia, Valencia, Spain

## Abstract

**Background:**

Non-surgical treatment of peri-implantitis includes a correct mechanical debridement of the implant surface to reduce the inflammation and recondition the soft tissues. The aim of the study was to evaluate the results of a single phase of non-surgical therapy by comparing the effect of curettes and ultrasounds versus curettes and abrasive air polisher (Air-Flow) in the peri-implant tissue conditions, and patient satisfaction.

**Material and Methods:**

A double-blind randomized and controlled prospective clinical study was conducted on patients in peri-implant maintenance phase diagnosed of peri-implantitis treated in the Oral Surgery Unit of the Stomatology Department of the Faculty of Medicine and Dentistry of the University of Valencia, between September of 2017 and May of 2018. They were divided into 2 groups: Group 1: curettes and ultrasounds, and Group 2: curettes and Air-Flow. The clinical and radiological baseline parameters were evaluated after 3-weeks of treatment, as well as patient satisfaction.

**Results:**

The sample included 34 patients. Group 1 (17 patients, 38 implants) and Group 2 (17 patients, 32 implants). All the variables improved statistically significantly after treatment in both groups, with the exception of recessions and keratinized mucosa and bone loss that did not vary. When comparing both groups, the type of treatment did not influence the majority of the variables, with the exception of the plaque index (*p*=0.011) and modified bleeding index from the palatine (*p*=0.048), which reduced statistically significant in the group 2, as well as the patient satisfaction which was higher in the group 2 (*p*<0.001).

**Conclusions:**

An initial phase of non-surgical treatment achieves an improvement of the peri-implant clinical parameters, thought the method of debridement used seems not to influence.

** Key words:**Peri-implantitis, peri-implant disease, non-surgical treatment, air-abrasive device, mechanical debridement.

## Introduction

Non-surgical treatment of peri-implantitis includes a correct mechanical debridement of the implant surface that will aim to reduce the inflammation of the peri-implant tissue (1). Although different methods of mechanical removal have been described in the literature there is no consensus regarding the method of decontamination to choose and remains a topic of discussion (2-4).

Recent reviews showed that the most commonly instruments used for implant surface debridement are curettes, ultrasonic devices and abrasive air polishers (5,6). Recent *in vitro* studies investigating implant debridement methods demonstrated that air-powder devices provided a superior cleaning potential compared to curettes or ultrasonic scalers (7). However, despite clinical studies have revealed significant improvements in probing depth, bleeding on probing, and microbiological tests when treating periimplantitis with glycine powder air-polishing (8-10) it is still argued that does not show superior results to other methods, such as manual curettes, ultrasonic scalers, and laser devices (9), and limited evidence regarding the effect of the non-surgical treatment in peri-implantitis on the improvement or stopping bone loss (11). On the other hand, the study of the degree of patient satisfaction with the treatment received is very important to determine if there is a greater degree of discomfort and to determine a greater preference towards a technique. Several studies on periodontal treatment found a lower degree of patient discomfort in the abrasive air polisher group compared to ultrasound (12,13); however, no study has been found on the non-surgical treatment of peri-implantitis. The efficacy of techniques such as abrasive air polisher or ultrasounds for the improvement of peri-implant clinical parameters has been widely studied and though, these methods were not found valid for the effective treatment of peri-implantitis, we hypothesize that the reconditioning of soft tissues prior to performing peri-implant surgery may improve tissue quality and the prognosis of surgical therapy.

The aim of this controlled study was to evaluate the effect of two mechanical methods of decontamination, curettes and ultrasounds versus curettes and abrasive air polisher, in the peri-implant health of patients with peri-implantitis. To this end, clinical parameters (plaque index, bleeding index, probing depth, clinical attachment, width of the keratinized mucosa, suppuration and recession) and radiological (bone loss) will be evaluated. A further aim was to assess the influence of the method of decontamination on patient’s satisfaction.

## Material and Methods

-Study design

A double-blind randomized and controlled prospective clinical trial was carried out on patients in peri-implant maintenance phase diagnosed of peri-implantitis in the Oral Surgery Unit of the Stomatology Department of the Faculty of Medicine and Dentistry of the University de Valencia, between September of 2017 and May of 2018. The design of the study was approved by the ethics committee of the Universitat de València (Ref: H1478032571959) and performed following the principles of the Declaration of Helsinki on human experimentation. All patients signed a consent to participate in the study, and to perform the peri-implant treatment.

-Selection Criteria

Patients with at least one implant diagnosed by peri-implantitis to the recent classification of peri-implant conditions (14), within the age range of 18 to 80 years, no presence of systemic disease or condition or medication known to alter bone metabolism (i.e. bisphosphonates) were included in the study. On the contrary, patients were excluded as study subjects if they presented a uncontrolled medical conditions such as diabetes mellitus, pregnancy, lactation, heavy smoking (≥20 cigarettes/day), implants that received a previous surgical treatment of peri-implantitis, or when was not possible to remove the prostheses and patients that failed to control visits.

-Randomization

A permuted block randomization approach was adopted to prepare the random number Tables and avoid imbalances between group. The patients were randomly assigned to one of the two study groups according to the treatment group (predictor variable): abrasive air polisher group (group 1: mechanical debridement of implants with curettes and abrasive air polisher with glicine powder) or ultrasounds group (group 2: mechanical debridement of implants with curettes and ultrasounds). Patients were blinded to the treatment allocation; however, due to the nature of the study, blinding of the operator was not possible. Radiological parameters were assessed by a two blinded investigators different from the operator. The procedure was also blinded for the principal investigator and statistician.

-Procedure

All patients that fulfilled inclusion and exclusion criteria were called to a non-surgical treatment. Prostheses were removed and local anesthesia Ultracain® (Normon, Madrid, Spain) was used. The prosthetic abutments such as abutments and screws were placed in an ultrasonic cell. In the case of the cemented prostheses, these were carefully lifted and all the cement residues removed. At this point, an assistant was asked to open a randomization envelope and the assigned treatment group technique was revealed and performed accordingly.

The non-surgical treatments were carried out by the second year students of the Master of Oral and Implantology Surgery, with the same degree of surgical experience. Mechanical debridement was performed in all cases with titanium curettes. The curettes were applied in a circular manner, running through each of the threads of the implant. Carbon curettes were also used to clean the surface of the polished neck. Abrasive air polisher was applied on each implant surface for 5 seconds as indicated by manufacturer´s guide (EMS Air-Flow Master Piezon® System (E.M.S. Electro Medical Systems S.A, Nyon, Switzerland). In both groups, 0.12% chlorhexidine was used by irrigation in the peri-implant sulcus. Finally, oral hygiene measures were reinforced. Chlorhexidine 0.12% rinses were prescribed 3 times a day for 15 days. Peri-implant soft tissue conditions were collected as a main variables; and bone loss and patient satisfaction as a secondary variables. The following parameters were registered before the treatment (T0) and 3-weeks after non-surgical treatment (T1).

-Data collected

Patient age (at implant placement), sex, frequency of brushing (≥ 3 times/day; 1-2 times/day), smoking habits (no smoking; < 10 cigarettes/day; 10-20 cigarettes/day), bruxism habit (yes, no), biotype, implant (brand, width, length, connection type), surgery date, and prosthesis design were registered.

Peri-implant soft tissue conditions: The plaque index (PI-score 0-3) and bleeding index (BI-score 0-3) were recorded following the guidelines put forward by Mombelli and Lang (22). Probing depth (PD, in mm) was assessed at four aspects (mesial, midfacial, distal, lingual/palatal) around each implant. The width of keratinized mucosa (WKM) and peri-implant facial mucosal retraction (level of the facial margin) were assessed on the midfacial aspect with a millimetered periodontal probe (Hu-Friedy UNC, Chicago, IL, USA).

Radiographic peri-implant marginal bone loss: Intraoral radiographs were obtained previous treatment (baseline) and after 3-weeks treatment using the XMIND intraoral system (GroupeSatelec-Pierre Rolland, Merignac, France) and an RVG intraoral digital receptor (Dürr Dental, Bietigheim-Bissingen, Germany) with the aid of Rinn XCP (DentsplyRinn, Elgin, IL, USA) to achieve parallelism. The images were calibrated with CliniView (version 5.1, Instrumentarium Imaging, Tuusula, Finland). The distance from the implant-abutment connection to the peri-implant marginal bone level was measured to the nearest 0.5 mm mesially and distally. Bone loss was calculated from the change in bone level between the baseline and 3-weeks follow-up radiograph.

Patient Satisfaction (or comfort level): The patient was asked if he would be willing to go through the non-surgical treatment of peri-implantitis again (yes / no), and about the level of comfort during mechanical debridement, ranging from 0 to 10.

All patients, after the initial single phase of non-surgical treatment, 3-weeks later, underwent the surgical procedure and were included in a monthly maintenance program.

-Statistical analysis

The primary outcome of the study was to evaluate the effect of the method of debridement on probing depth. A value of 0.7 mm was used for the sample size calculation and a SD of 0.7. These values were chosen due to the lack of evidence published in the Literature about this topic, to the best of our knowledge. The statistical power for this test was 80.8% to detect an effect of 0.7 with a confidence of 95% and alpha set at 0.05.

Continuous variables were described by the number of observations (n), minimum (min), median, maximum (max), mean, and standard deviation (SD) values and discrete variables by frequencies and percentages. Within-group and between-group comparisons were calculated using Chi2, Mann-Whitney test, regression lineal, and Friedman test. A *p*-value <.05 was considered as being statistically significant. In the study of the error, two intraobserver and one interobserver tests were performed. The Dahlberg d was used to measure random error and the intraclass correlation coefficient (ICC) was used to assess the random error. The degree of reproducibility was high in both intra- and interexaminations (ICC=0.97 and 0.94, respectively).

## Results

During the study period, 38 patients participated in the study. Four dropouts occurred during the observation period. A total of 34 patients (18 men and 16 women, mean age of 58.4±9.9 years with a range of 41–86 years) were treated with 70 implants: Group 1, 17 patients, and Group 2, 17 patients. The mean number of implants that patients presented in the mouth was 5.15 ± 3.2 (range 1-12). The mean number of implants with peri-implantitis was 2.06 ± 1.01 (range 1-5). The study groups were homogeneous with respect to all variables studied.

-Analytical study of peri-implant soft tissues

All variables improved statistically significantly after treatment in both groups, except for the presence of recessions (*p* = 0.705) and its length (buccal *p* = 0.605, palatal *p* = 0.718) and the keratinized mucosa (*p* = 0.134) which did not vary after the 3-weeks of treatment (Table 1).

**Table 1:**
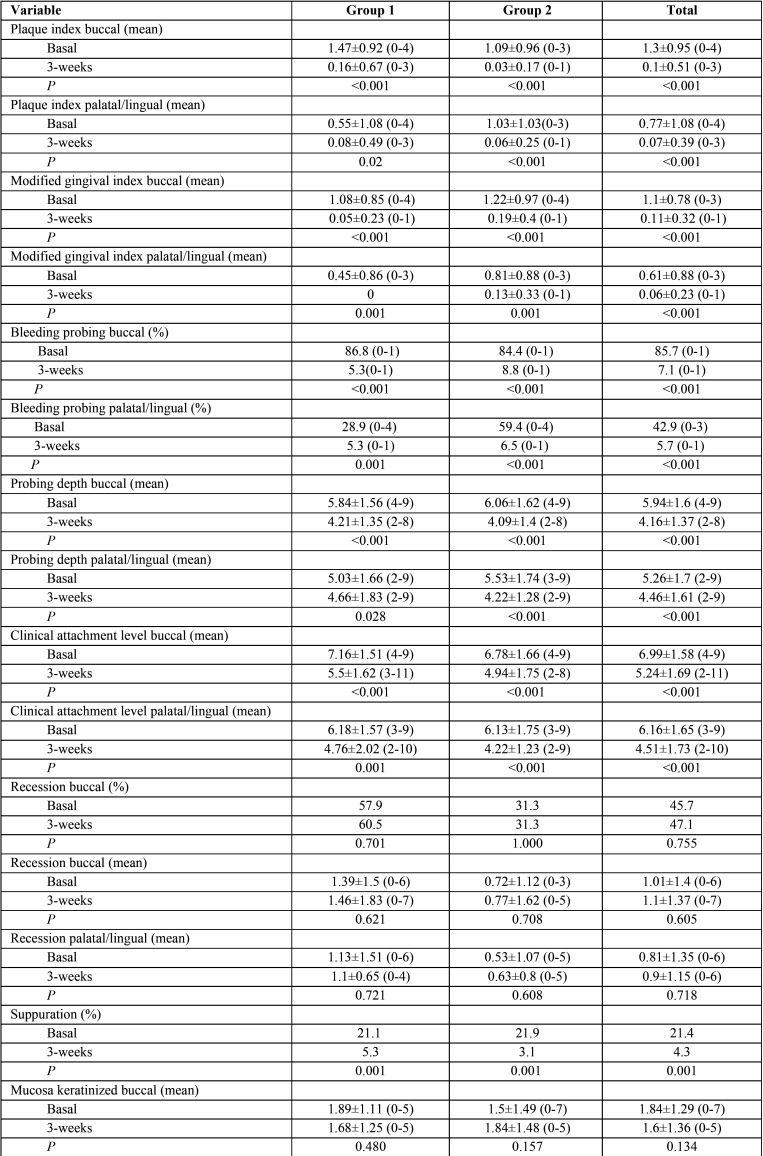
Descriptive and analytical data of changes on peri-implant soft tissues at baseline and 3-weeks after treatment.

When comparing both treatments, it was observed that in group 2 palatal plaque and bleeding rates decreased more than in group 1 being this difference statistically significant. For the rest of the variables studied, no statistically significant differences were found (Table 2).

**Table 2:**
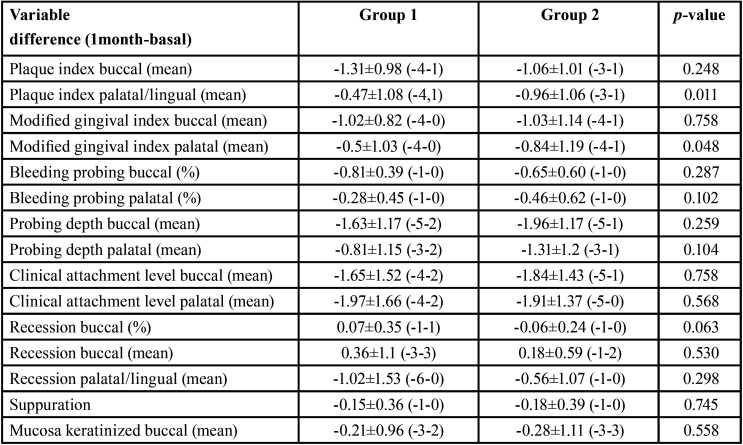
Descriptive and analytical data of the effect of the treatment received between groups on peri-implant soft tissue conditions.

-Analytical study of peri-implant marginal bone loss

There were no statistically significant changes in bone loss between the baseline measurements and during the follow-up period (*p* = 0.903). No differences were found between groups (Table 3).

**Table 3:**
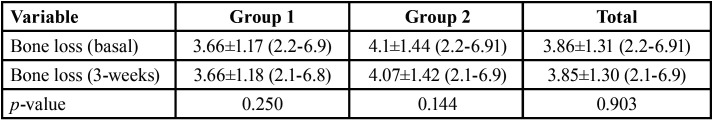
Descriptive and analytical data of changes on mean bone loss between groups.

-Analytical study of patient satisfaction

All patients in both groups reported that they would be willing to go through the same procedure again. The mean overall satisfaction of the patient with the treatment was 8.5±7.05 (range 6-9). Patient satisfaction level in group 1 was 7±0.61 and group 2 was 8±0.5 (*p*<0.001).

## Discussion

The purpose of this randomized prospective study was to compare the effect of two mechanical methods of decontamination, curettes and ultrasounds versus curettes and abrasive air polisher, in the peri-implant conditions of implants with peri-implantitis. The results of the present investigation demonstrated that after performing the non-surgical treatment all the clinical variables improved but the debridement method did not influence the outcomes.

In the 6th Periodontics Workshop it was concluded that there is no standard and effective treatment for the treatment of peri-implantitis (15). The different types of study design, as well as the different population samples and different methods made difficult to compare and extrapolate the results. In the present study in order not to alter the results, all measurements were assessed by the same operator previously trained to perform measurements reliably. The homogeneity of the randomized groups was evaluated with respect to all studied variables.

Recent systematic reviews aimed at assessing the efficacy of non-surgical treatment procedures for the management of peri-implant mucositis and peri-implantitis revealed that clinical improvements can be gained following a non-surgical treatment in peri-implant mucositis, (1,9,10,16-18); though it is however difficult to achieve healthy soft tissues completely free from clinical signs of inflammation (19). However, in peri-implantitis, non-surgical techniques were considered not to be effective (18,20). Persson *et al.* (20) compared mechanical treatment with curettes versus ultrasonic device and demonstrated that the clinical changes in both probing depht and bleeding on probing between baseline and 6 months after treatment suggested limited clinical improvements with no treatment group differences. Recent systematic reviews about the treatment of peri-implantitis found that mechanical submucosal debridement alone had very limited effect on the clinical signs of peri-implantitis (18) and the adjunctive chlorhexidine application could improve the effect, though with limited outcomes (18,21). John *et al.* (22) at 6- and 12 months following non-surgical treatment of peri-implantitis using a mechanical debridement in adjuction with local antiseptic therapy of chlorhexidine digluconate obtained significant lower probing of depth reductions, though failed to achieve a complete disease resolution (23,24). The systematic review conducted by Swartz *et al.* (17) did not identify any superior effect of air polishing (i.e. adjunctive use and monotherapy) over mechanical debridement in either reducing clinical signs of inflammation or obtaining disease resolution at mucositis sites (10,25), though an improved efficacy of glycine powder air polishing in reducing bledding on probing scores after nonsurgical treatment of peri-implantitis was found over the control (26). However, a tendency towards a re-infection over time has been showed after treatment (16). Based on the available data, it seems that the nonsurgical therapy of peri-implantitis is not effective in disease resolution because only limited improvements in the main clinical parameters have been reported and there is a clear tendency for disease recurrence and advanced therapies, such as surgical interventions should to be considered when nonsurgical peri-implant surgery is unable to achieve signiﬁcant improvements in the clinical parameters (11).

In the present study all clinical variables improved without differing between the treatment modality, though the peri-implant bone loss did not change, similarly as found in the revised literature (11,18,27). Mutukuru *et al* (1) reported limited information available about the progression of bone loss in peri-implantitis following non-surgical treatment.

Few studies have evaluated the degree of patient satisfaction in peri-implantitis according to the method of mechanical debridement used (curettes, ultrasound or abrasive air polisher) (Ji). Ji *et al.* (26) conducted a pilot clinical trial evaluating the effect of the abrasive air polisher with glycine in the treatment of peri-implant mucositis with respect to a control group (curettes); no complication or discomfort was reported by patients. In the present study patients who underwent Air-Flow therapy scored statistically higher degree of satisfaction. The fact that patients scored better on the Air-Flow treatment with respect to the ultrasound group could be due to the lower noise and vibration produced by the device. It is also important to consider the key limiting factors in these studies, such as the reduced sample sizes or the lack of a control group using only curettes.

In periimplantitis, non-surgical treatment should be addressed to infection control through debridement of the implant surface with the aims of debriding the adhered bioﬁlm and reducing the bacterial load below the threshold level for causing disease (11). That is why the main outcomes were clinical peri-implant variables as the aim of the study was to evidence the improvement of tissue quality evidenced by a reduction of clinical inflammation signs after the non-surgical treatment, to demonstrate the importance of performing an initial phase of decontamination and debridement before starting a surgical procedure in implants affected by peri-implantitis. This initial phase achieves reducing inflammation that may permit the tissues to face the surgical procedure in better conditions. Future investigation should focus on the need of performing an initial session of non-surgical treatment before surgical procedures on peri-implantitis to improve clinical parameters to perform the posterior surgical treatment on healthier peri-implant tissues more than to apply the non-surgical treatment as an isolated option in disease resolution.
